# Supplementary material for: Medial temporal lobe atrophy patterns in early- versus late-onset amnestic Alzheimer’s disease
Source: bioRxiv. 2024 May 21:2024.05.21.594976. Preprint. [Version 1] doi: 10.1101/2024.05.21.594976 (PMC11142072; doi:10.1101/2024.05.21.594976)
Supplement: Supplement 2 [file media-2.pdf]

Supplementary Results
Atrophy patterns and co-pathologies in early- versus late-onset amnestic Alzheimer's disease

Table of Contents

Amnestic EOAD shows medial temporal lobe subfield involvement **2**

sTable 3. 2
sFigure 11 2
sTable 4. 3

Further characterization of amnestic EO- and LOAD **4**

Neocortical thickness differences in EO- vs. LOAD *4*
sFigure 12 4

LEADS signature thickness and tau-PET uptake group comparisons *5*
sFigure 13. 5

Differences in co-pathologies in EO- vs. LOAD *6*
sTable 5. 6
sTable 6. 6
sFigure 14 7
sFigure 15. 7

Associations between (co-)pathologies and structural measures within amnestic EOAD *8*
sFigure 16 8

Cognitive performance in amnestic EOAD *9*
sTable 7. 9
sFigure 17. 10

Comparison between amnestic and non-amnestic EO- and LOAD *11*
Demographics 11
sTable 8. 11
sTable 9 12
sTable 10 13
sFigure 18 14
sFigure 19 15
sFigure 20 16

Amnestic EOAD shows medial temporal lobe subfield involvement

sTable 3. Comparison between groups on structural MRI measures.

	YCU vs. EOAD			OCU vs. LOAD			EOAD vs. LOAD	
	diff	p_{FDR}	p_{FDR} age adjusted	diff	p_{FDR}	p_{FDR} age adjusted	diff	p_{FDR}
**SUB**	1.617	**<.001**	**<.001**	1.249	**<.001**	**<.001**	0.502	**0.004**
**DG**	1.043	**<.001**	**<.001**	0.993	**<.001**	**<.001**	0.380	**0.042**
**CA1**	1.124	**<.001**	**<.001**	1.122	**<.001**	**<.001**	0.387	**0.042**
**ERC**	0.808	**<.001**	**<.001**	1.598	**<.001**	**<.001**	0.779	**0.003**
**BA35**	1.698	**<.001**	**<.001**	1.196	**<.001**	**<.001**	0.409	0.058
**BA36**	0.608	**<.001**	**<.001**	0.601	**<.001**	**<.001**	0.337	0.060
**PHC**	1.035	**<.001**	**<.001**	0.913	**<.001**	**<.001**	0.677	**<.001**
**Total HC**	1.684	**<.001**	**<.001**	1.547	**<.001**	**<.001**	0.499	**0.011**
**AMY**	1.899	**<.001**	**<.001**	1.548	**<.001**	**<.001**	0.366	0.147
**LT**	1.314	**<.001**	**<.001**	1.036	**<.001**	**<.001**	0.381	**0.031**
**LP**	1.536	**<.001**	**<.001**	0.754	**<.001**	**<.001**	-0.114	0.583
**MP**	1.439	**<.001**	**<.001**	0.849	**<.001**	**<.001**	0.049	0.741
**FL**	0.510	**.001**	**.002**	0.689	**<.001**	**<.001**	0.461	**0.014**
**OL**	0.371	**.034**	**.037**	0.276	**.029**	**.009**	0.157	0.438

Positive mean differences indicate higher values in the group listed first; negative mean differences indicate lower values in the group listed first. All analyses were adjusted for sex. All p-values are FDR adjusted.
Abbreviations: AMY=amygdala; BA=Brodmann area; CA1=cornu ammonis 1; DG=dentate gyrus; diff=mean difference; ERC=entorhinal cortex; EOAD=amnestic early-onset cognitive impairment; FL=frontal cortex; HC=hippocampus; LOAD=amnestic late-onset cognitive impairment; LT=lateral temporal; LP=lateral parietal; MP=medial parietal; naEOAD=non-amnestic early-onset AD; naLOAD=non-amnestic late-onset AD; OCU=older cognitively unimpaired controls; OL=occipital cortex; PHC=parahippocampal cortex; SUB=subiculum; YCU=younger cognitively unimpaired controls.

sFigure 11. Mean differences for the comparisons between EOAD with controls and LOAD with controls showing similar differences across regions.

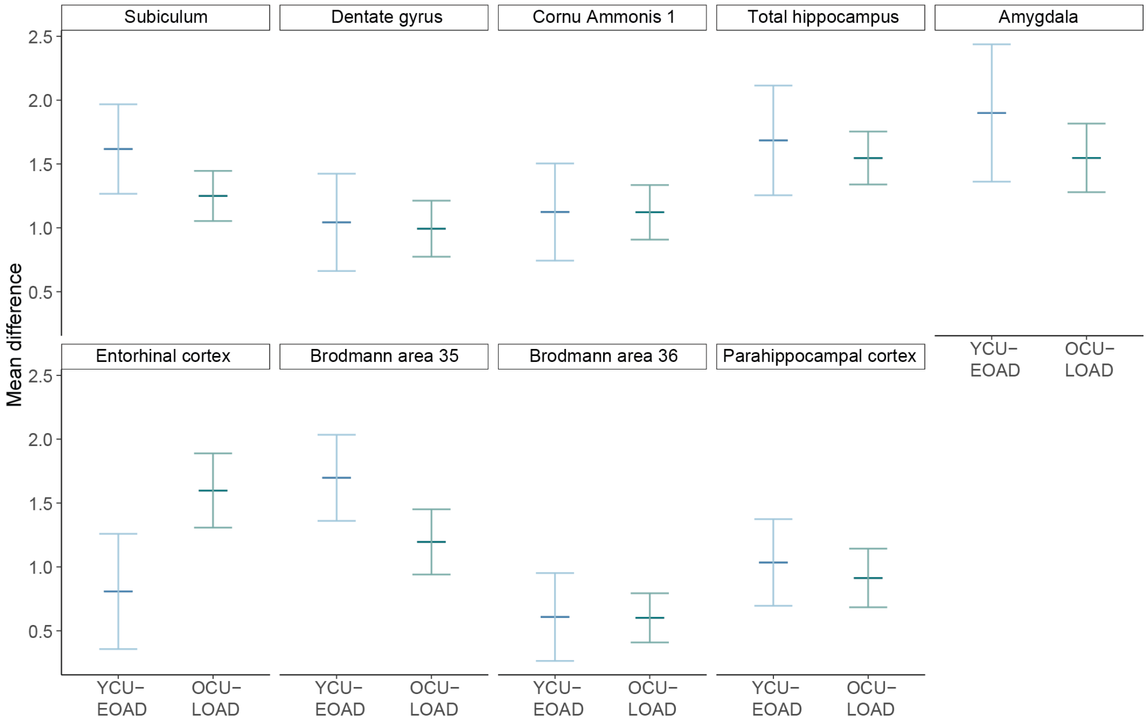

Mean differences in the groups are shown with a 95%-confidence interval. Abbreviations: AMY=amygdala; BA=Brodmann Area; CA1=Cornu Ammonis 1; DG=dentate gyrus; EOAD=early-onset Alzheimer's Disease; ERC=entorhinal cortex; FL=frontal cortexHC=total hippocampus; LOAD=late-onset Alzheimer's disease; LT=lateral temporal cortex; LP=lateral parietal cortex; MP=medial parietal cortex; OCU=older controls; OL=occipital cortex; PHC=parahippocampal cortex; SUB=subiculum; YCU=younger controls.

sTable 4. Results of the interaction analyses between age (young/old) and diagnosis (CU/AD) for all regions of interest.

MTL subfields	Std. beta	95%-CI		p_{FDR}
**SUB**	0.134	(-0.003	0.730)	0.157
**DG**	0.017	(-0.378	0.464)	0.977
**CA1**	-0.002	(-0.400	0.389)	0.977
**ERC**	-0.262	(-1,323	-0.333)	**0.010**
**BA35**	0.163	(0.059	0.922)	0.117
**BA36**	-0.011	(-0.371	0.323)	0.977
**PHC**	0.049	(-0.274	0.533)	0.792
**Total HC**	0.046	(-0.253	0.518)	0.792
**AMY**	0.104	(-0.138	0.824)	0.363

The results show only the interaction term for each region of interest. The interaction between analyses were adjusted for sex. All p-values are FDR adjusted.

Abbreviations: AMY=amygdala; BA=Brodmann area; CA1=cornu ammonis 1; DG=dentate gyrus; diff=mean difference; ERC=entorhinal cortex; EOAD=amnestic early-onset cognitive impairment; HC=hippocampus; LOAD=amnestic late-onset cognitive impairmentOCU=older cognitively unimpaired controls; PHC=parahippocampal cortex; SUB=subiculum; YCU=younger cognitively unimpaired controls.

Further characterization of amnestic EO- and LOAD

Neocortical thickness differences in EO- vs. LOAD

sFigure 12. EO- vs. LOAD group differences in neocortical volume/thickness.

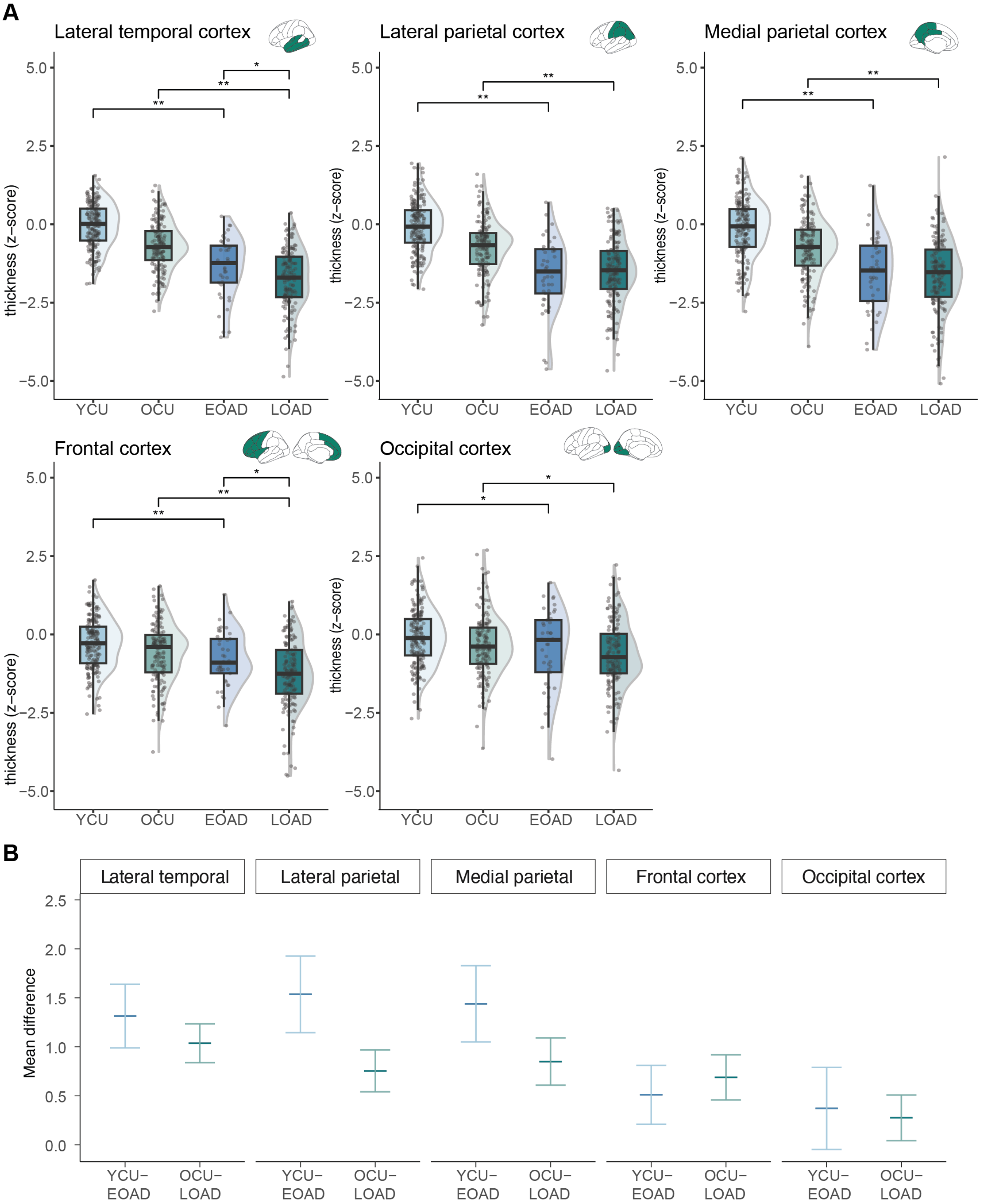

Interaction										
Std. beta	0.121	p=0.155	0.330	**p<.001**	0.230	**p=0.010**	-0.070	p=0.516	0.041	p=0.653
95%-CI	-0.049 - 0.628		0.436 - 1.172		0.197 - 1.025		-0.536 - 0.220		-0.317 - 0.505	

A shows the group comparisons. ANOVAs were performed for each comparison. See sTable 6 for more information. **B** shows the mean differences of the comparisons of the AD groups with respective controls and the results of the interaction analysis (age*diagnosis) for the interaction term. Significant differences are shown for FDR-corrected p-values. Abbreviations: EOAD=early-onset Alzheimer's Disease; LOAD=late-onset Alzheimer's disease; OCU=older controls; YCU=younger controls.

LEADS signature thickness and tau-PET uptake group comparisons

sFigure 13. Group comparison for LEADS signature thickness and tau-PET uptake.

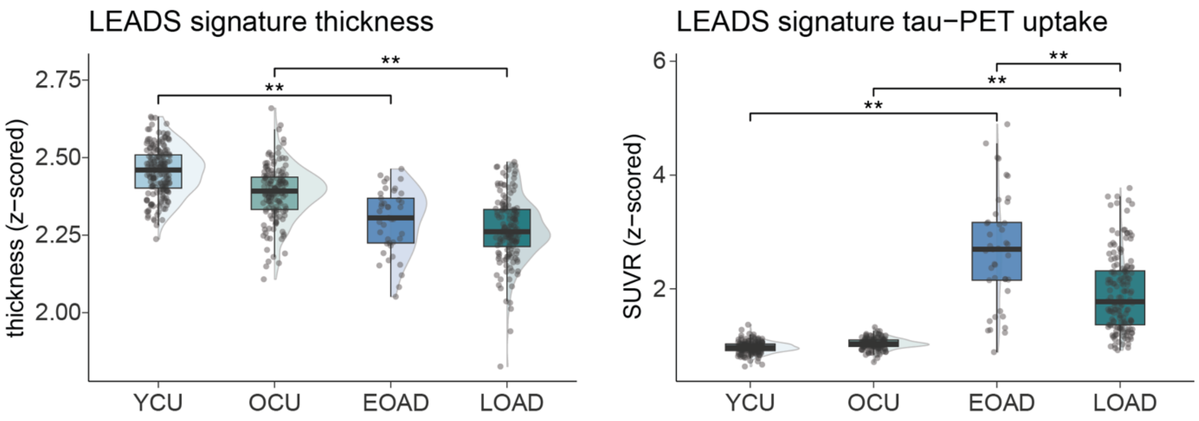

Significant differences are shown for FDR-corrected p-values for the exploratory analyses investigating the LEADS signature for thickness and tau-PET uptake. LEADS signature based on Touroutoglou et al. (2023).
Abbreviations: EOAD=early-onset Alzheimer's Disease; LOAD=late-onset Alzheimer's disease; LEADS=Longitudinal Early-Onset Alzheimer's Disease Study; PET=positron emission tomography; SUVR=standardized uptake value ratio; OCU=older controls; YCU=younger controls.

Both EO- and LOAD showed significantly thinner thickness compared to controls (p<0.001, 95%-C.I.=[-0.20, -0.12]; p<0.001, 95%-C.I.=[-0.14, -.08] respectively). No differences between EO- and LOAD were observed (p=.351, 95%-C.I.=[0.02, 0.35]).

Both EO- and LOAD showed significantly higher tau-PET uptake compared to controls (p<0.001, 95%-C.I.=[1.49, 1.91]; p<0.001, 95%-C.I.=[0.75, 1.02] respectively). Additionally, EOAD showed a significantly higher tau-PET uptake compared to LOAD (p<0.001, 95%-C.I.=[-0.96, -0.54]).

Differences in co-pathologies in EO- vs. LOAD

sTable 5. AD pathologies, co-pathologies, and cognitive measures of the sample.

	YCU	OCU	EOAD	LOAD	Total	pFDR YCU-EOAD	pFDR OCU-LOAD	pFDR EOAD-LOAD
**N**	188	151	41	154	534	-	-	-
**CSF Aβ42/40 ratio**	1.02±0.13	0.99±0.14	0.46±0.09	0.47±0.11	0.81±0.29	**<.001**	**<.001**	.655
**MTL tau-PET**	1.02±0.26	1.21±0.26	2.67±0.59	2.67±0.65	1.67±0.87	**<.001**	**<.001**	.975
**Amygdala tau-PET**	0.82±0.11	0.87±0.11	2.23±0.67	2.08±0.61	1.31±0.72	**<.001**	**<.001**	.207
**EBM-II tau-PET**	1.04±0.10	1.10±0.10	2.57±0.92	2.18±0.73	1.51±0.76	**<.001**	**<.001**	**.006**
**EBM-III tau-PET**	0.99±0.10	1.07±0.11	3.01±1.25	1.84±0.64	1.42±0.77	**<.001**	**<.001**	**<.001**
**EBM-IV tau-PET**	0.88±0.10	0.93±0.10	1.68±0.69	1.31±0.41	1.08±0.40	**<.001**	**<.001**	**<.001**
**EBM-V tau-PET**	0.94±0.09	1.00±0.10	1.61±0.61	1.24±0.30	1.10±0.32	**<.001**	**<.001**	**<.001**
**WMH vol.**	3320±2200	8140±6020	5670±3720	9690±6950	6690±5830	**<.001**	.085	**<.001**
**WMH vol.** dich.	13 (6.9)	69 (45.7)	11 (26.8)	80 (51.9)	173 (32.4)	**<.001**	.277	**.004**
**aHC/PHC ratio +**	23 (12.2)	3 (2.0)	15 (36.6)	44 (28.6)	85 (15.9)	**<.001**	**<.001**	.333

Continuous variables are displayed as mean±SD. Categorical variables are displayed as n (%). P-values are FDR adjusted. Aβ positivity: <.08 on CSF Aβ42/40 ratio.

Abbreviations: Aβ=amyloid-beta; aHC=anterior hippocampus; CSF=cerebrospinal fluid; dich=dichotomized variable; EBM=event-based modeling; EOAD=early-onset cognitive impairment; FDR=false-discovery rate adjusted p-values; HC=Hippocampus; ROI=region of interest; LOAD=late-onset cognitive impairment; OCU=older cognitively unimpaired controls; PHC=parahippocampal cortex; SD=standard deviation; YCU=younger cognitively unimpaired controls; WMH=white matter hyperintensities.

sTable 6. AD pathologies, co-pathologies, and cognitive measures of the sample with comparisons between controls and AD groups adjusted for age.

	YCU	OCU	EOAD	LOAD	pFDR YCU-EOAD	pFDR OCU-LOAD
**N**	188	151	41	154	-	-
**CSF Aβ42/40 ratio**	1.02±0.13	0.99±0.14	0.46±0.09	0.47±0.11	**<.001**	**<.001**
**MTL tau-PET**	1.02±0.26	1.21±0.26	2.67±0.59	2.67±0.65	**<.001**	**<.001**
**Amygdala tau-PET**	0.82±0.11	0.87±0.11	2.23±0.67	2.08±0.61	**<.001**	**<.001**
**EBM-II tau-PET**	1.04±0.10	1.10±0.10	2.57±0.92	2.18±0.73	**<.001**	**<.001**
**EBM-III tau-PET**	0.99±0.10	1.07±0.11	3.01±1.25	1.84±0.64	**<.001**	**<.001**
**EBM-IV tau-PET**	0.88±0.10	0.93±0.10	1.68±0.69	1.31±0.41	**<.001**	**<.001**
**EBM-V tau-PET**	0.94±0.09	1.00±0.10	1.61±0.61	1.24±0.30	**<.001**	**<.001**
**WMH vol.**	3320±2200	8140±6020	5670±3720	9690±6950	**<.001**	.033
**WMH vol.** dich.	13 (6.9)	69 (45.7)	11 (26.8)	80 (51.9)	**<.001**	.262
**aHC/PHC ratio +**	23 (12.2)	3 (2.0)	15 (36.6)	44 (28.6)	**<.001**	**<.001**

Continuous variables are displayed as mean±SD. Categorical variables are displayed as n (%). P-values are FDR adjusted. Aβ positivity: <.08 on CSF Aβ42/40 ratio.

Abbreviations: Aβ=amyloid-beta; aHC=anterior hippocampus; CSF=cerebrospinal fluid; dich=dichotomized variable; EOAD=early-onset cognitive impairment, ROI=region of interest; LOAD=late-onset cognitive impairment; OCU=older cognitively unimpaired controls; PHC=parahippocampal cortex; SD=standard deviation; YCU=younger cognitively unimpaired controls.

sFigure 14. Group comparison for the dichotomized white matter hyperintensity volumes.

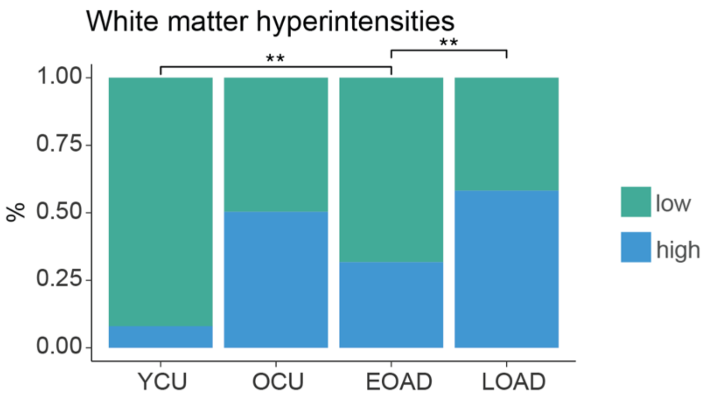

Significant differences are shown for FDR-corrected p-values.
Abbreviations: EOAD=early-onset Alzheimer's Disease; LOAD=late-onset Alzheimer's disease; OCU=older controls; YCU=younger controls.

sFigure 15. Group comparison for tau-PET uptake in neocortical composite regions.

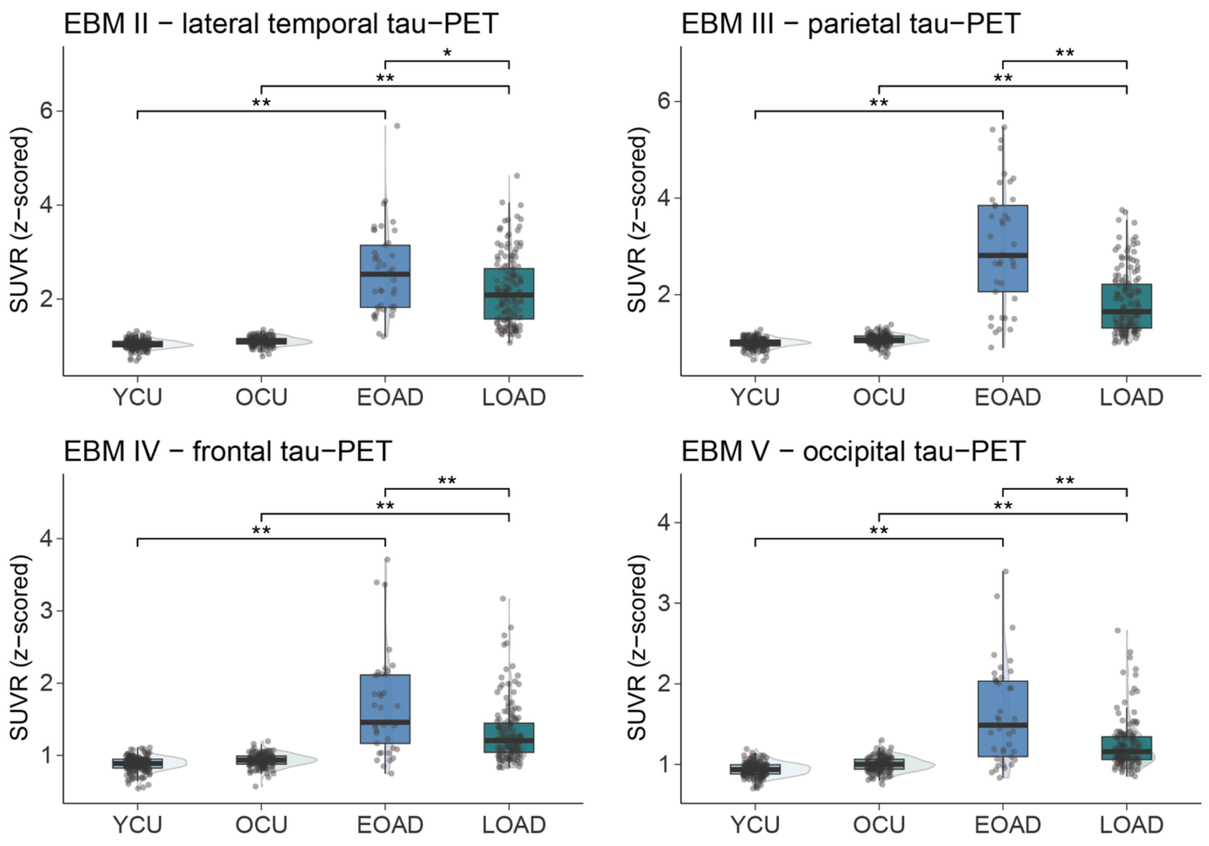

Separate ANOVAs were performed for each comparison. Significant differences are shown for FDR-corrected p-values. A: additional group comparisons for neocortical tau-PET uptake and dichotomized white matter hyperintensity volume. B: exploratory analyses investigating the LEADS signature for thickness and tau-PET uptake. LEADS signature based on Touroutoglou et al. (2023).
Abbreviations: EOAD=early-onset Alzheimer's Disease; LOAD=late-onset Alzheimer's disease; LEADS=Longitudinal Early-Onset Alzheimer's Disease Study; PET=positron emission tomography; SUVR=standardized uptake value ratio; OCU=older controls; YCU=younger controls.

Associations between (co-)pathologies and structural measures within amnestic EOAD

sFigure 16. Associations between (co-)pathologies of interest with structural measures.

A

	tau-PET SUVR	CSF Aβ42/40	WMH vol	aHC/pHC ratio
Total Hippocampus	0.06	0.08	0.16	−0.63
Entorhinal cortex	−0.15	−0.14	−0.01	−0.33
Parahippocampal cortex	0.06	−0.19	−0.24	0.16

(Colored cell: p<.05)

B

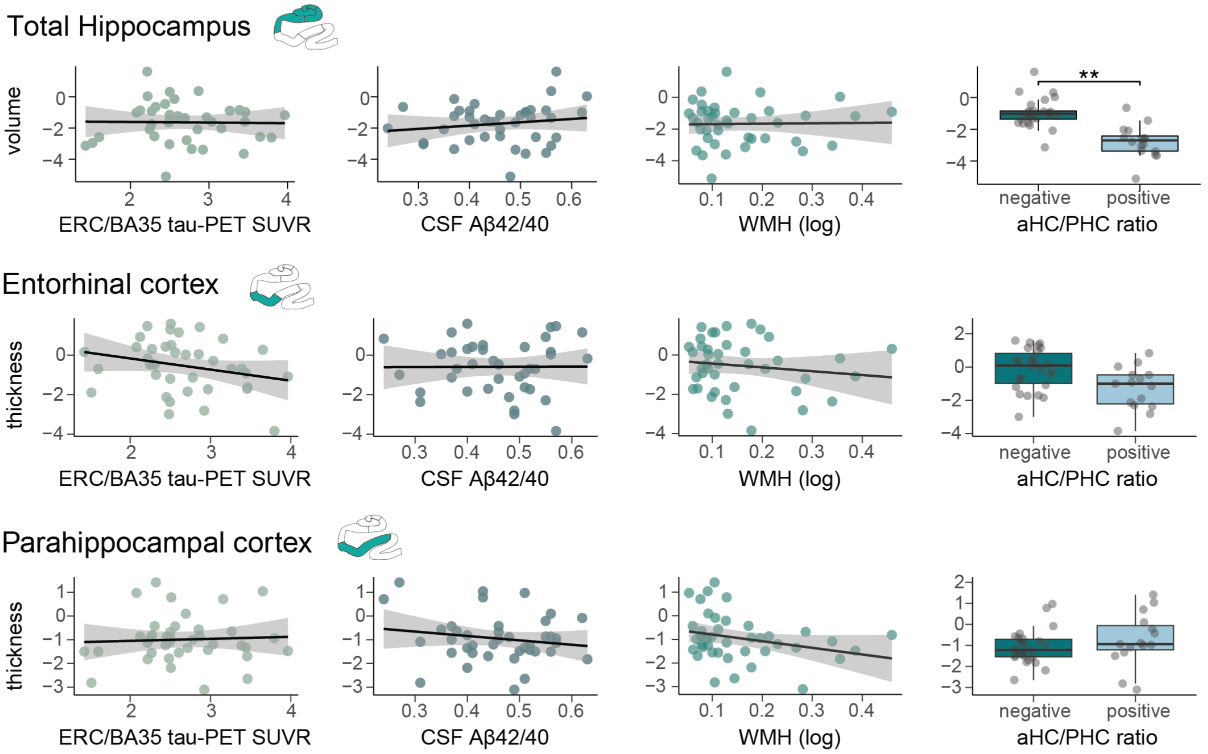

Exploratory linear regressions were performed focusing on EOAD only. A: shows the standardized beta coefficients of the linear regression models. Significant associations are shown with FDR-corrected p-values (colored cells are p_{FDR}<.05). B: shows scatterplots and boxplots of the investigated associations.

Abbreviations: BA35=Brodmann area 35; CSF=cerebrospinal fluid; EOAD=early-onset Alzheimer's Disease; ERC=entorhinal cortex; LOAD=late-onset Alzheimer's disease; NEG=negative; PET=positron emission tomography; POS=positive; SUVR=standardized uptake value ratio; OCU=older controls; YCU=younger controls.

Cognitive performance in amnestic EOAD

sTable 7. Comparisons of cognitive performance across the groups.

	YCU	OCU	EOAD	LOAD	Total	p-value YCU-EOAD	p-value OCU-LOAD	p-value EOAD-LOAD
**N**	188	151	41	154	534	-	-	-
**ADAS delayed**	1.89±1.49	2.98±1.82	8.02±1.44	8.53±1.45	4.59±3.34	**<.001**	**<.001**	.291
**Animal fluency**	26.3±5.74	23.0±4.99	17.2±5.37	13.9±4.85	21.1±7.36	**<.001**	**<.001**	**.004**
**BNT-15**	14.3±0.99	13.7±1.42	12.7±2.56	10.9±2.83	13.0±2.40	**<.001**	**<.001**	**<.001**
**VOSP cube**	9.73±0.67	9.56±0.98	7.84±2.85	8.41±2.14	9.18±1.65	**<.001**	**<.001**	.197
**SDM**	48.3±9.31	36.7±7.93	26.5±12.9	24.6±8.14	36.9±13.2	**<.001**	**<.001**	.662
**TMT-B**	70.4±24.4	100±45.3	227±139	250±124	138±111	**<.001**	**<.001**	.466

Continuous variables are displayed as mean±SD. Categorical variables are displayed as n (%). [a] individuals who reported an age-of-onset under 65 were included in the EOAD group. [b] Aβ positivity: <.08 on CSF Aβ42/40 ratio.

Abbreviations: Aβ=amyloid-beta; CSF=cerebrospinal fluid; EOAD=early-onset cognitive impairment, LOAD=late-onset cognitive impairment; OCU=older cognitively unimpaired controls; SD=standard deviation; YCU=younger cognitively unimpaired controls.

sFigure 17. Associations between all cognitive test scores with structural MRI measures.

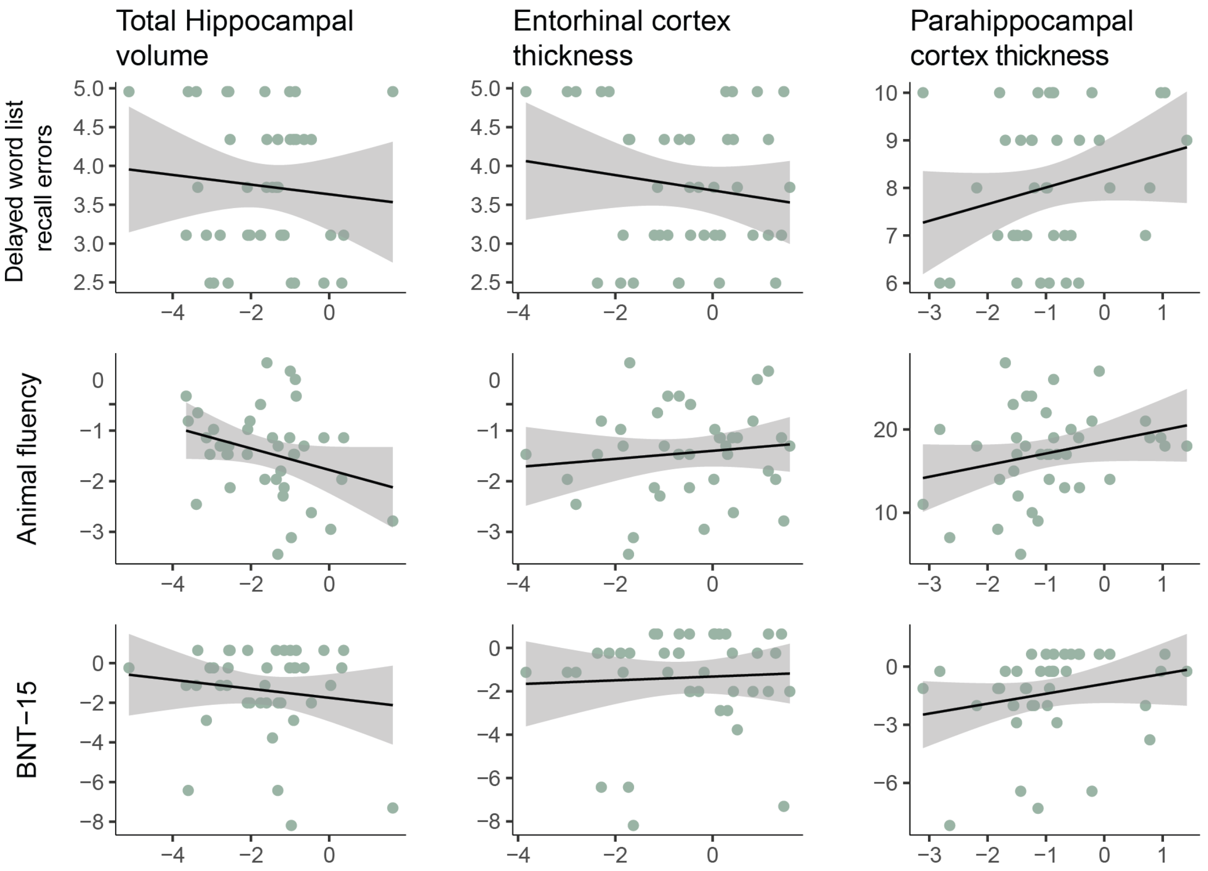

Linear regressions were performed using only the EOAD group (n=41) and including age, sex, and education as covariates. None of the associations were statistically significant.

Comparison between amnestic and non-amnestic EO- and LOAD

Demographics

sTable 8. Characteristics of the sample including non-amnestic Alzheimer's disease cases.

	YCU	OCU	EOAD	naEOAD	LOAD	naLOAD	Total
**N**	188	151	41	7	154	16	557
**Diagnosis** (CU/MCI/AD)	188/0/0	151/0/0	0/16/25	0/3/4	0/65/89	0/10/6	339/95/123
**Sex** (female)	103 (54.8)	99 (65.5)	20 (48.8)	3 (42.9)	82 (53.2)	7 (43.8)	314 (56.4)
**Age**	58.6±4.89	77.3±3.38	61.0±4.82	61.6±3.98	76.2±3.92	77.4±3.98	69.3±9.72
Range	51.0 – 69.0	70.3 – 85.0	50.9 – 69.4[a]	56.0 – 66.1	70.1 – 85.1	71.3 – 85.4	50.9 – 85.4
**Education** (years)	13.2±3.12	12.4±3.74	14.1±3.33	13.6±2.82	12.5±4.79	12.8±4.09	12.8±3.85
Missing	2 (1.1)	0 (0.0)	1 (2.4)	0 (0.0)	6 (3.9)	0 (0.0)	9 (1.6)
***APOE-ε4* allele**[b]	85 (45.2)	29 (19.2)	25 (61.0)	5 (71.4)	114 (74.0)	9 (56.3)	267 (47.9)
**CSF Aβ42/40 +**	0 (0.0)	0 (0.0)	41 (100)	7 (100)	154 (100)	16 (100)	218 (39.1)

Continuous variables are displayed as mean±SD. Categorical variables are displayed as n (%). [a] Aβ positivity: <.08 on CSF Aβ42/40 ratio. Abbreviations: Aβ=amyloid-beta; aHC=anterior hippocampus; CSF=cerebrospinal fluid; EOAD=amnestic early-onset cognitive impairment; ROI=region of interest; LOAD=amnestic late-onset cognitive impairment; naEOAD=non-amnestic early-onset AD; naLOAD=non-amnestic late-onset AD; OCU=older cognitively unimpaired controls; PHC=parahippocampal cortex; SD=standard deviation; YCU=younger cognitively unimpaired controls.

sTable 9. Comparison between groups on structural MRI measures including non-amnestic AD groups.

	**YCU vs. EOAD**		**YCU vs. naEOAD**		**OCU vs. LOAD**		**OCU vs. naLOAD**		**EOAD vs. LOAD**		**naEOAD vs. naLOAD**		**EOAD vs. naEOAD**		**LOAD vs. naLOAD**	
	diff	p_{FDR}	diff	p_{FDR}	diff	p_{FDR}	diff	p_{FDR}	diff	p_{FDR}	diff	p_{FDR}	diff	p_{FDR}	diff	p_{FDR}
SUB	1.617	**<.001**	0.454	0.215	1.249	**<.001**	1.136	**<.001**	0.502	**0.008**	1.551	**<.001**	-1.162	**0.011**	-0.113	0.722
DG	1.043	**<.001**	0.378	0.419	0.993	**<.001**	0.674	**0.022**	0.380	0.066	0.726	0.154	-0.665	0.201	-0.319	0.305
CA1	1.124	**<.001**	0.049	0.877	1.122	**<.001**	0.509	0.065	0.387	0.066	0.849	0.107	-1.075	**0.041**	-0.613	**0.041**
ERC	0.808	**<.001**	-0.235	0.804	1.598	**<.001**	0.438	**0.093**	0.779	**0.006**	0.662	0.397	-1.043	0.109	-1.160	**0.007**
BA35	1.698	**<.001**	0.644	0.121	1.196	**<.001**	0.827	**0.003**	0.409	0.088	1.095	0.072	-1.054	**0.022**	-0.368	0.355
BA36	0.608	**<.001**	0.272	0.411	0.601	**<.001**	0.464	**0.014**	0.337	0.092	0.536	0.263	-0.336	0.543	-0.138	0.793
PHC	1.035	**<.001**	0.887	**0.039**	0.913	**<.001**	0.991	**<.001**	0.677	**0.001**	0.902	0.112	-0.148	0.793	0.077	0.882
Total HC	1.684	**<.001**	0.369	0.373	1.547	**<.001**	0.775	**0.002**	0.499	**0.020**	1.043	0.055	-1.316	**0.037**	-0.772	**0.010**
AMY	1.899	**<.001**	0.404	0.341	1.548	**<.001**	0.892	**0.002**	0.366	0.207	1.205	0.072	-1.495	**0.048**	-0.656	0.094
LT	1.314	**<.001**	1.061	**0.001**	1.036	**<.001**	1.013	**<.001**	0.381	**0.048**	0.612	0.280	-0.254	0.603	-0.023	0.902
LP	1.536	**<.001**	1.470	**0.000**	0.754	**<.001**	0.825	**0.006**	-0.114	0.676	0.022	0.947	-0.066	0.888	0.070	0.888
MP	1.439	**<.001**	1.249	**0.003**	0.849	**<.001**	0.776	**0.014**	0.049	0.804	0.166	0.798	-0.190	0.782	-0.073	0.793
FL	0.510	**0.002**	0.454	0.263	0.689	**<.001**	0.826	**0.008**	0.461	**0.023**	0.654	0.227	-0.056	0.877	0.137	0.804
OL	0.371	0.054	0.047	0.892	0.276	**0.044**	0.190	0.551	0.157	0.521	0.395	0.504	-0.324	0.646	-0.086	0.804

Positive mean differences indicate higher values in the group listed first; negative mean differences indicate lower values in the group listed first. All analyses were adjusted for sex. All p-values are FDR adjusted.
Abbreviations: aHC=anterior hippocampus; AMY=amygdala; BA=Brodmann area; CA1=cornu ammonis 1; DG=dentate gyrus; diff=mean difference; ERC=entorhinal cortex; EOAD=amnestic early-onset cognitive impairment; FL=frontal cortex; HC=hippocampus; LOAD=amnestic late-onset cognitive impairment; LT=lateral temporal; LP=lateral parietal; MP=medial parietal; naEOAD=non-amnestic early-onset AD; naLOAD=non-amnestic late-onset AD; OCU=older cognitively unimpaired controls; OL=occipital cortex; PHC=parahippocampal cortex; SUB=subiculum; YCU=younger cognitively unimpaired controls.

sTable 10. Comparison between groups on AD biomarkers and co-pathologies and cognitive measures.

	YCU-EOAD		YCU-naEOAD		OCU-LOAD		OCU-naLOAD		EOAD-LOAD		naEOAD-naLOAD		EOAD-naEOAD		LOAD-naLOAD	
	diff	p_{FDR}	diff	p_{FDR}	diff	p_{FDR}	diff	p_{FDR}	diff	p_{FDR}	diff	p_{FDR}	diff	p_{FDR}	diff	p_{FDR}
CSF Aβ42/40	0.561	**<.001**	0.533	**<.001**	0.527	**<.001**	0.575	**<.001**	-0.009	.727	0.067	.170	-0.028	.638	0.048	.161
ERC/BA35 tau-PET SUVR	-1.646	**<.001**	-1.177	**<.001**	-1.455	**<.001**	-1.168	**<.001**	0.001	.982	-0.182	.623	0.469	.148	0.286	.182
EBM II tau-PET SUVR	-1.538	**<.001**	-1.255	**<.001**	-1.081	**<.001**	-1.045	**<.001**	0.390	**.008**	0.144	.831	0.283	.601	0.036	.919
EBM III tau-PET SUVR	-2.011	**<.001**	-1.399	**<.001**	-0.772	**<.001**	-0.740	**<.001**	1.167	**<.001**	0.586	.346	0.612	.365	0.032	.923
EBM IV tau-PET SUVR	-0.799	**<.001**	-0.634	**<.001**	-0.382	**<.001**	-0.484	**<.001**	0.366	**<.001**	0.099	.892	0.165	.669	-0.102	.558
EBM V tau-PET SUVR	-0.676	**<.001**	-0.489	**<.001**	-0.242	**<.001**	-0.243	**<.001**	0.371	**<.001**	0.183	.553	0.187	.601	-0.001	.919
Amygdala tau-PET SUVR	-1.404	**<.001**	-0.673	**<.001**	-1.206	**<.001**	-0.624	**<.001**	0.148	.270	-0.001	.998	0.731	**.032**	0.582	**.002**
WMH volume	-0.065	**<.001**	-0.052	.063	-0.041	.120	-0.163	**.001**	-0.111	**<.001**	-0.247	.061	0.013	.828	-0.123	**.034**
Delayed word list recall	-3.780	**<.001**	-1.563	**<.001**	-3.423	**<.001**	-1.014	**.003**	-0.313	.176	-0.121	.638	2.217	**<.001**	2.409	**<.001**
Animal fluency	1.497	**<.001**	1.362	**.001**	1.498	**<.001**	1.610	**<.001**	0.544	**.001**	0.791	.182	-0.135	.842	0.112	.663
BNT-15	1.411	**<.001**	1.925	**<.001**	2.481	**<.001**	1.837	**<.001**	1.616	**.004**	0.457	.842	0.514	.727	-0.644	.545
VOSP Cube	2.935	**<.001**	1.441	**.010**	1.783	**<.001**	2.220	**<.001**	-0.896	.148	1.035	.736	-1.493	.566	0.437	.638
Symbol Digit Modalities	1.815	**<.001**	2.111	**<.001**	1.017	**<.001**	1.165	**<.001**	0.164	.553	0.017	.924	0.296	.540	0.148	.566
TMT-B	-4.385	**<.001**	-4.752	**<.001**	-4.189	**<.001**	-4.378	**<.001**	-0.643	.727	-0.465	.831	-0.367	.878	-0.189	.895
	OR	p_{FDR}	OR	p_{FDR}	OR	p_{FDR}	OR	p_{FDR}	OR	p_{FDR}	OR	p_{FDR}	OR	p_{FDR}	OR	p_{FDR}
aHC/PHC ratio +	4.575	**<.001**	3.121	.288	20.073	**<.001**	2.658	.553	0.685	.437	0.143	.274	0.716	.801	5.637	.161

All analyses were adjusted for sex. All p-values are FDR adjusted.

Abbreviations: BA35=Brodmann area 35; BNT-15=Boston Naming Test-15; diff=mean difference; ERC=entorhinal cortex; EOAD=amnestic early-onset cognitive impairment; LOAD=amnestic late-onset cognitive impairment; naEOAD=non-amnestic early-onset AD; naLOAD=non-amnestic late-onset AD; OCU=older cognitively unimpaired controls; OR=odds ratui; YCU=younger cognitively unimpaired controls.

sFigure 18. Boxplots showing significant differences of comparisons with the non-amnestic and amnestic AD groups for medial temporal lobe structural MRI measures.

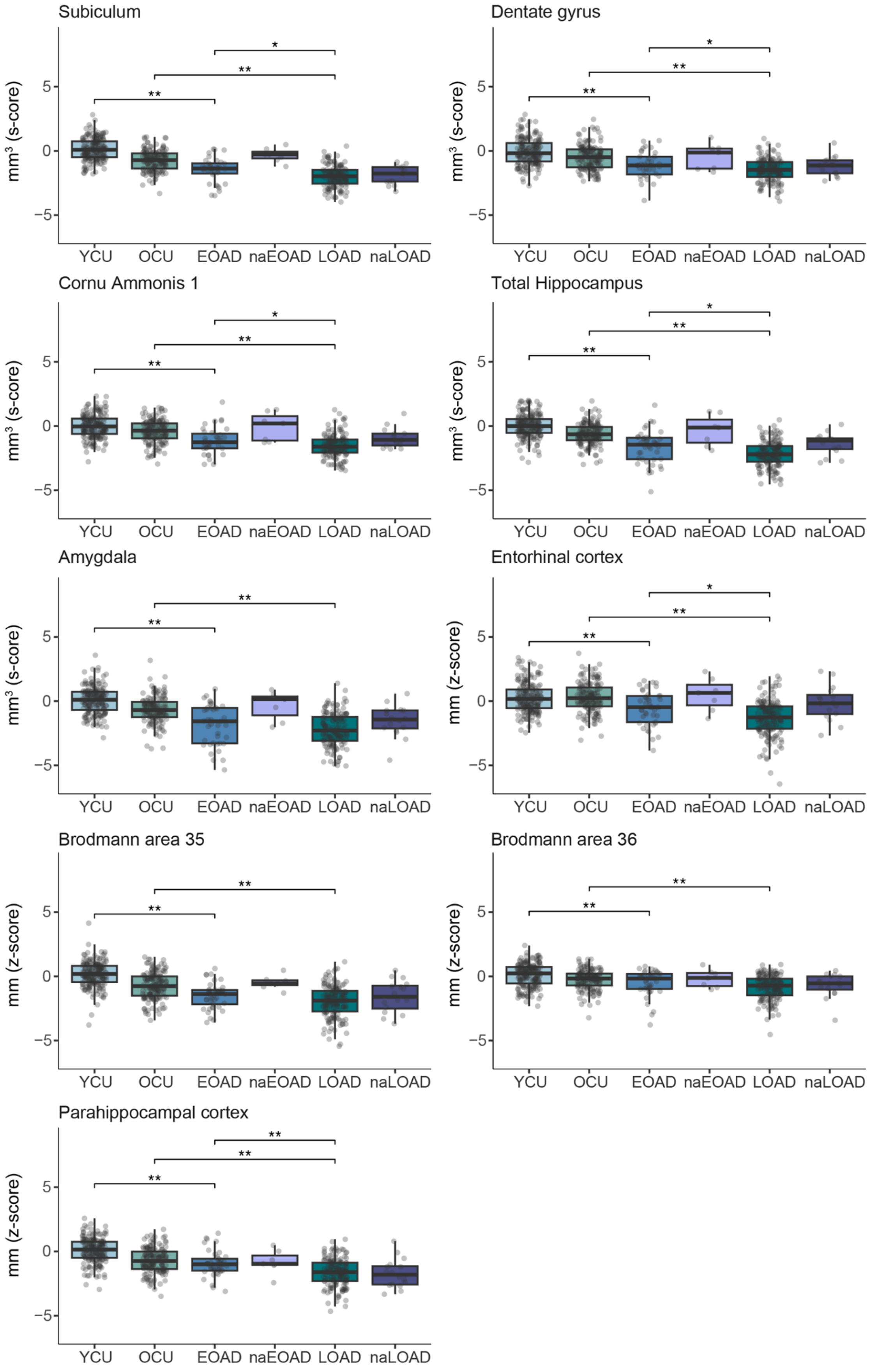

ANOVAs were performed for each comparison. Significant differences are shown for FDR-corrected p-values. Abbreviations: EOAD=amnestic early-onset Alzheimer's Disease; LOAD=amnestic late-onset Alzheimer's disease; naEOAD=non-amnestic early-onset AD; naLOAD=non-amnestic late-onset AD; OCU=older controls; YCU=younger controls.

sFigure 19. Boxplots showing significant differences of comparisons with the non-amnestic and amnestic AD groups for the neocortical structural measures.

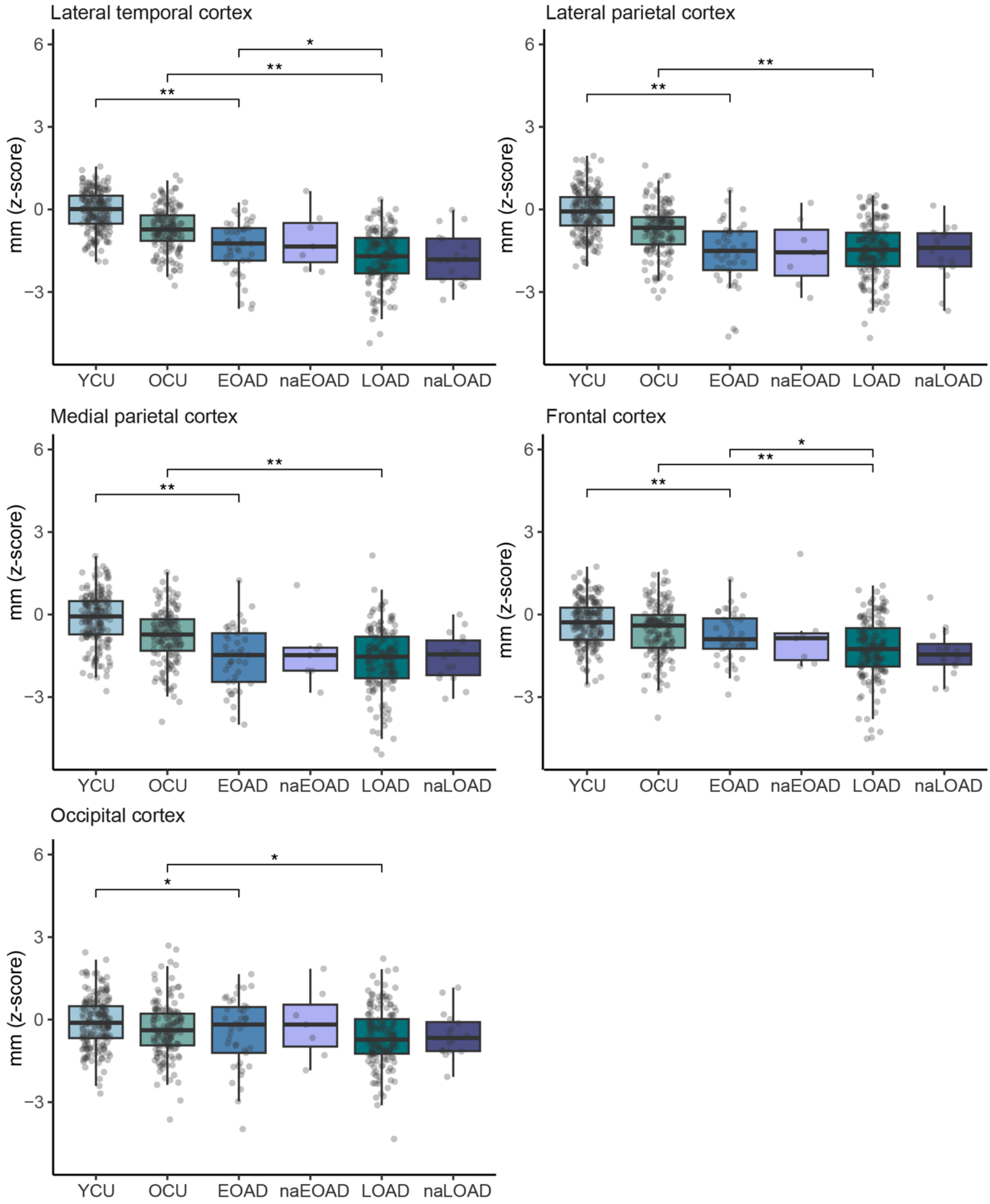

ANOVAs were performed for each comparison. Significant differences are shown for FDR-corrected p-values. Abbreviations: EOAD=amnestic early-onset Alzheimer's Disease; LOAD=amnestic late-onset Alzheimer's disease; naEOAD=non-amnestic early-onset AD; naLOAD=non-amnestic late-onset AD; OCU=older controls; YCU=younger controls.

sFigure 20. Boxplots showing significant differences of comparisons with the non-amnestic and amnestic AD groups for the significant pathologies and cognitive measures.

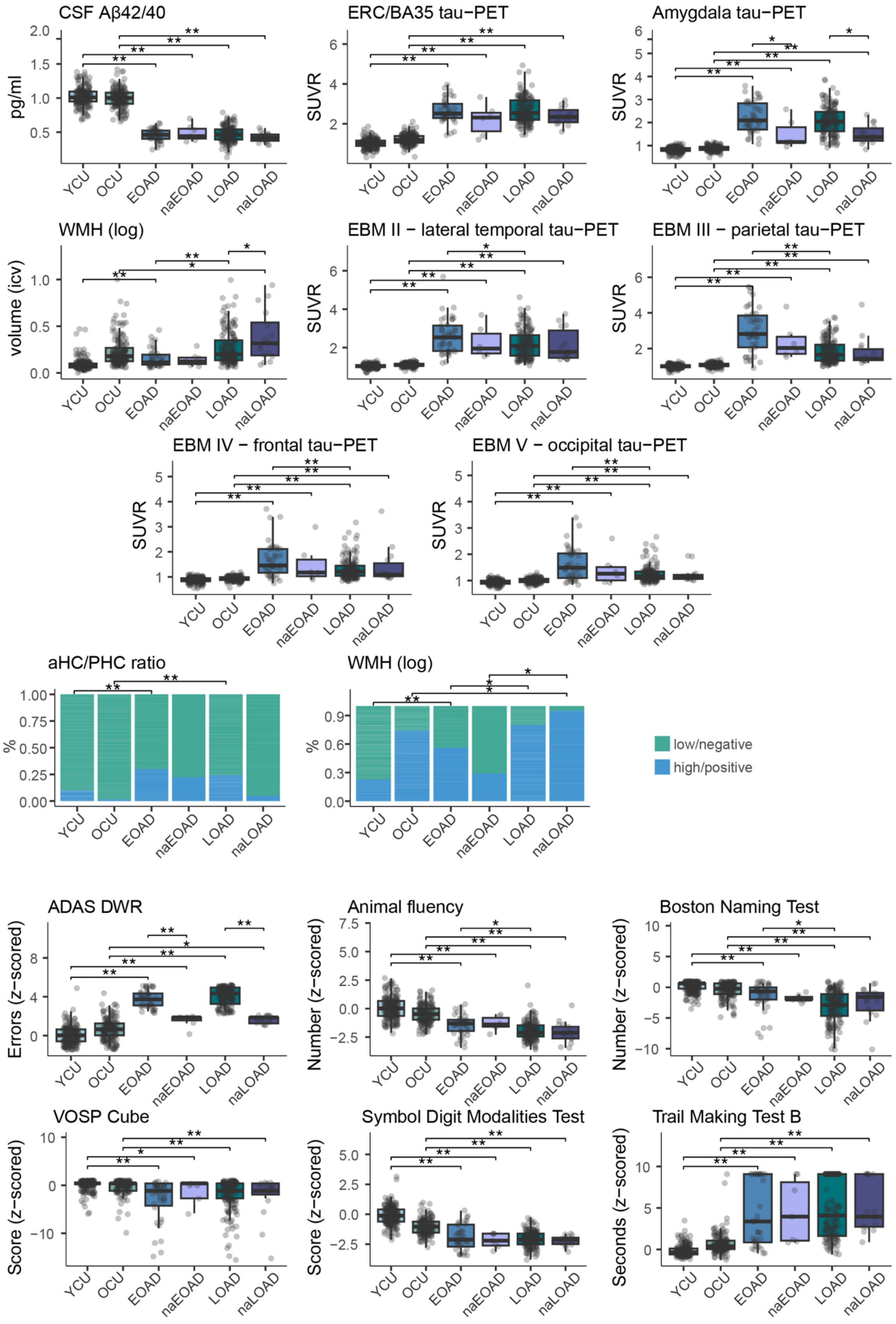

ANOVAs were performed for each comparison. Significant differences are shown for FDR-corrected p-values. Abbreviations: EOAD=amnestic early-onset Alzheimer's Disease; LOAD=amnestic late-onset Alzheimer's disease; naEOAD=non-amnestic early-onset AD; naLOAD=non-amnestic late-onset AD; OCU=older controls; YCU=younger controls.
